# Effect of neoadjuvant chemotherapy regimen on relapse-free survival among patients with breast cancer achieving a pathologic complete response: an early step in the de-escalation of neoadjuvant chemotherapy

**DOI:** 10.1186/s13058-018-0945-7

**Published:** 2018-04-16

**Authors:** Anna Weiss, Sami I. Bashour, Kenneth Hess, Alastair M. Thompson, Nuhad K. Ibrahim

**Affiliations:** 1 Department of Surgical Oncology, Brigham and Women’s Hospital, Dana Farber Cancer Institute, Boston, MA 02115 USA; 2 Department of Breast Medical Oncology, University of Texas MD Anderson Cancer Center, 1155 Pressler Street CPB5.3540, Houston, TX 77030 USA; 3 Department of Biostatistics, University of Texas MD Anderson Cancer Center, Houston, TX 77030 USA; 4 Department of Breast Surgical Oncology, University of Texas MD Anderson Cancer Center, Houston, TX 77030 USA

**Keywords:** Neoadjuvant chemotherapy, Pathologic complete response, De-escalation of chemotherapy, Breast cancer, Survival after pCR

## Abstract

**Background:**

Patients with breast cancer who have a pathologic complete response (pCR) to neoadjuvant chemotherapy **(**NACT) have improved survival. We hypothesize that once pCR has been achieved, there is no difference in subsequent postsurgical recurrence-free survival (RFS), whichever NACT regimen is used.

**Methods:**

Data from patients with breast cancer who achieved pCR after NACT between 1996 and 2011 were reviewed. RFS was estimated by the Kaplan-Meier method, and differences between groups were assessed using log-rank testing. Cox proportional hazards regression analysis adjusted for age, menopausal status, stage, grade, tumor subtype, and adjuvant endocrine HER2-targeted radiation treatment.

**Results:**

Among 721 patients who achieved pCR after NACT, 157 (21.8%) were hormone receptor-positive (HR), 310 (43.3%) were HER2-amplified, and 236 (32.7%) were triple-negative; 292 (40.5%) were stage IIA, 153 (21.2%) were stage IIB, 78 (10.8%) were stage IIIA, 66 (9.2%) were stage IIIB, and 132 (18.3%) were stage IIIC. Most patients (367 [50.9%]) had been treated with adriamycin-based chemotherapy plus taxane (A + T), 56 (7.8%) without taxane (A no T), 227 (31.5%) with HER2-targeted therapy, and 71 (9.8%) provider choice. Median follow-up was 7.1 years. Adjuvant chemotherapy was employed in 196 (27%) patients, adjuvant endocrine in 261 (36%), and adjuvant radiation in the majority (559 [77.5%]). There was no statistically significant difference in RFS by NACT group. Adjusted RFS hazard ratios, comparing each treatment with the reference group A + T, were 1.25 (95% CI 0.47–3.35) for A no T, 0.90 (95% CI 0.37–2.20) for HER2-targeted therapy, and 1.28 (95% CI 0.55–2.98) for provider choice.

**Conclusions:**

These data suggest that postsurgical RFS is not significantly influenced by the choice of NACT or cancer subtype among patients achieving pCR.

**Electronic supplementary material:**

The online version of this article (10.1186/s13058-018-0945-7) contains supplementary material, which is available to authorized users.

## Background

Neoadjuvant chemotherapy (NACT), originally used to downstage breast cancer, may achieve a pathologic complete response (pCR) in a proportion of patients, with no residual invasive disease in the breast or nodes [1]. The residual cancer burden (RCB) present after NACT reflects response to chemotherapy and predicts survival. Consequently, low (RCB I) or no residual disease (RCB 0, defined as pCR) is associated with improved survival compared with more residual disease (RCB II or III), a relationship that holds true across breast cancer subtypes [1–4]. Large randomized clinical trials have shown that more recent NACT regimens have provided increased pCR rates [5, 6], and some trials have shown resultant improved survival [1, 7]. Although the timing of chemotherapy and use of NACT have not historically impacted overall survival (OS) [8–13], important therapeutic information may be gained by evaluating tumor response to chemotherapy [14, 15]. Although an imperfect tool [1], pCR has been approved by the U.S. Food and Drug Administration as an acceptable surrogate endpoint for key clinical trials [16–19].

The relationship between complete pathologic response to NACT and survival in triple-negative breast cancer has been used for statistical modeling and clinical trial power calculations [20]. This modeling assumes that all patients with a pCR have similar survival, but this assumption of NACT regimen equivalence has not been examined. The objectives of this study were to determine if a specific treatment regimen may be associated with preferential survival in patients who achieve a pCR and to identify risk factors for recurrence among patients with a pCR.

## Methods

Patients diagnosed with primary breast cancer on the basis of cytology or histopathology needle biopsy, treated between 1996 and 2011 with various regimens of NACT at a single high-volume cancer center, were reviewed using a prospectively maintained electronic database. Patients were considered eligible for review if, after NACT, they achieved pCR on the basis of final surgical pathology. pCR in this study was defined as eradication of invasive disease in both the breast and lymph nodes (T0/Tis, N0), the definition used at our institution, consistent with the large pooled analysis performed by Cortazar et al. [1]. Patients were excluded if any part of their chemotherapy regimen was received at an outside institution or after definitive breast surgery. This single-institution study was approved by the University of Texas MD Anderson Cancer Center Institutional Review Board.

Patients were divided into the following four major chemotherapy regimens: adriamycin-based chemotherapy alone (A no T), adriamycin-based chemotherapy plus taxane (A + T), HER2-targeted therapy (AT + HER2), or provider choice (PC). Provider choice regimens were all others; that is, all included a taxane base, but with the addition of clinical trial agents such as capecitabine or bevacizumab. Duration of NACT was calculated as the date of first chemotherapy treatment to the date of surgery, including a typical 4- to 6-week window between completion of NACT and surgery. There were three NACT duration cohorts: ≤ 4 months, > 4 and ≤ 7 months, and > 7 months. The group treated for ≤ 4 months was treated early in the study time frame according to a departmental algorithm that included four cycles only (most commonly doxorubicin, cyclophosphamide, and 5-fluorouracil; or fluorouracil, adriamycin, and cyclophosphamide [FAC]). Recurrence-free survival (RFS) of pCR patients was compared between these four regimens, agnostic to the details of drugs received. Recurrence and death data were collected by in-depth chart review. The RFS was estimated by the Kaplan-Meier method; calculations were made at 5 years; and the differences between groups were assessed using log-rank testing. Values were statistically significant if *P* < 0.05. RFS was next compared by breast cancer tumor subtype (hormone receptor [HR]-positive, HER2-amplified, and triple-negative [TN]), and then within each of these subtypes the RFS was again calculated and compared for the four specific NACT regimens. RFS was next calculated by age group (21–40, 41–60, and > 60 years old), stage (IIA, IIB, IIIA, IIIB, and IIIC), and grade (1–3). Age groups were determined by quartiles, with patients aged 21–40 years constituting the youngest quartile and those aged > 60 years the oldest quartile [21]. Last, the RFS was compared by length of neoadjuvant chemotherapy.

Adjuvant treatments were analyzed via landmark analysis [22] with time point zero set to 6 months following surgery to allow for completion of adjuvant treatments (adjuvant chemotherapy, radiation). The landmark cutoff did not include completion of endocrine or anti-HER2 therapy. Patients who were censored or failed (recurrence or death within 6 months of surgery) were omitted. This approach allowed adjuvant treatments to be included as baseline factors in the analysis. Patients omitted were those who failed as described (*n* = 4) or if clinicopathologic details (age, grade, stage, HR or HER2 status, adjuvant treatments received) were missing (*n* = 75). Too few patients had neoadjuvant radiation treatment (*n* = 1) or neoadjuvant endocrine treatment (*n* = 6) for landmark analysis. Cox proportional hazards regression analysis adjusted for NACT (using treatment arm A + T as the reference group), age (reference group 41- to 60-year-olds), menopausal status (reference group premenopausal), stage (reference group stage IIA), grade (reference group grade 1), tumor subtype (reference group TN breast cancer), and adjuvant treatments (reference group for each therapy was no treatment received).

## Results

### Patient characteristics

**Fig. 1:**
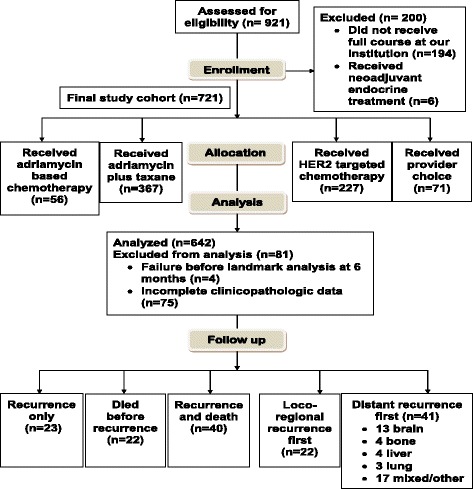
Consolidated Standards of Reporting Trials (CONSORT) diagram depicting the entire study cohort. There were 921 patients with a pathologic complete response at our institution during the study period. Two hundred of these patients received part of their chemotherapy at an outside institution. A total of 721 patients were included in survival analyses, and 642 in were included the multivariate analysis

A total of 921 patients were identified over the 15-year period, 200 of whom received part of their chemotherapy treatment at an outside institution and were excluded, leaving 721 patients in the survival analyses (Fig. 1). Of these 721 patients, 664 (92%) had ductal histology (Table 1). The youngest quartile of age, 21–40 years, included 154 patients (21.4%), the group ages 41–60 years included 449 patients (62.3), and the group aged > 60 years included 118 patients (16.4%). Race breakdown included 423 (59%) white patients, 136 (19%) Hispanic patients, 109 (15%) black patients, 45 (6%) Asian patients, and 8 (1%) categorized as unknown/other. A majority of patients (389 [54%]) were postmenopausal. Most patients 292 (40.5%) were stage IIA, 153 (21.2%) were stage IIB, 78 (10.8%) were stage IIIA, 66 (9.2%) were stage IIIB, and 132 (18.3%) were stage IIIC. By tumor subtype, 157 (21.8%) were HR-positive, 310 (43.3%) were HER2-amplified, 236 (32.7%) were TN, and 18 (2.5%) were of unknown type. Of the 310 HER2-amplified patients, 128 were HR+, 178 were HR−, and 4 had unknown HER2 status. Patients were grouped into four chemotherapy regimens: 367 (50.9%) patients treated with A + T, 56 (7.8%) treated with A no T, 227 (31.5%) treated with AT + HER2, and 71 (9.8%) treated with PC (Fig. 1). HER2+ patients were treated both before and after the standard use of HER2-targeted therapies, 247 received HER2-targeted therapy (20 of these in the PC group), and 60 did not. Survival was also examined within this subset. The median length of NACT was 6.0 months, ranging from < 1 month to 12 months. The majority of patients with shorter duration of NACT (< 4 months) received four cycles of FAC or fluorouracil, epirubicin, and cyclophosphamide (43 of 64 [67%]). Other regimens included adriamycin plus a taxane (10 of 64 [16%]); taxane alone (6 of 64; [9%]); FAC + taxane (3 of 64; [5%]); adriamycin and cyclophosphamide (1 of 64; [2%]); and taxotere, carboplatin, and herceptin (1 of 64 [2%]). In this group, 61 of 64 patients (95%) completed their planned regimen without issue. Two patients stopped NACT after three cycles because of neutropenia, and one patient proceeded to surgery after only two cycles per the patient’s request.

**Table 1:** Patient characteristics of the entire cohort

Characteristics	No. of patients (*N* = 721)	%
Breast cancer type		
Invasive ductal	664	92
Invasive lobular	9	1
Mixed ductal/lobular	14	2
Breast cancer, NOS^a^	16	2
Other	18	3
Age, years		
21–40	154	21
41–60	449	63
> 60	118	16
Race		
White	423	59
Hispanic	136	19
Black	109	15
Asian	45	6
Other	8	1
Menopausal status		
Unknown	1	0
Premenopausal	314	44
Pregnant	3	0
Perimenopausal	14	2
Postmenopausal	389	54
Clinical stage		
IIA	292	41
IIB	153	21
IIIA	78	11
IIIB	66	9
IIIC	132	19
Estrogen receptor status		
Unknown	15	2
Negative	454	63
Positive	252	35
Progesterone receptor status		
Unknown	19	3
Negative	526	73
Positive	176	24
HER2 status		
Negative	286	40
Positive	312	43
Equivocal	123	17
Tumor subtype		
Unknown	18	3
HR	157	22
TN	236	33
HER2-amplified	310	43
HR+	128	41
HR−	178	57
Unknown HR	4	1
Neoadjuvant chemotherapy		
A no T	56	8
A + T	367	51
AT + HER2	227	31
PC	71	10
HER2+ patient regimens		
HER2-containing regimen	247	80
No HER2	60	20
Length of NACT therapy		
0–4 months	64	9
4 & 7 months	570	79
7 months	87	12
Neoadjuvant hormonal therapy		
Yes	12	0
No	715	100
Neoadjuvant radiation therapy		
Yes	1	0
No	720	100
Adjuvant chemotherapy		
Yes	196	27
No	525	73
Adjuvant endocrine therapy		
Yes	261	36
No	460	64
Adjuvant radiation therapy		
Yes	559	78
No	162	22
Outcomes		
Progression	63	9
Death (any cause)	62	9
Progression + death	40	6
Death without progression	22	3
Site of metastasis		
Distant first	41	65
Brain-only	13	32
Bone-only	4	10
Liver-only	4	10
Lung-only	3	7
Mixed/other	17	41
Local first	11	17
Chest wall-only	1	9
Ipsilateral breast-only	10	91
Both local and distant	11	17
Concurrent^a^	6	55
Sequential	5	45

*Abbreviations: NOS* Not otherwise specified, *HR* Hormone receptor; *TN* Triple-negative breast cancer, *A no T* Adriamycin-based therapy alone, *A + T* Adriamycin plus taxane, *AT + HER2* HER2-targeted therapy, *PC* Provider choice, *NACT* Neoadjuvant chemotherapy

^a^Concurrent local and distant metastases were diagnosed within 1 month of each other

Definitive surgical treatments included total mastectomy and axillary lymph node dissection (ALND) in 256 (35.5%), total mastectomy and sentinel lymph node biopsy (SLNB) in 46 (6.4%), skin-sparing mastectomy and ALND in 44 (6.1%), skin-sparing mastectomy and SLNB in 51 (7.1%), partial mastectomy and ALND in 176 (24.4%), partial mastectomy and SLNB in 130 (18%), 1 radical mastectomy (0.1%), 2 wide local excisions after breast conservation (0.3%), 5 excisional biopsies (0.7%), and 10 ALND only (1.4%). Most patients (708 [98.2%]) had free surgical margins at initial operation. Adjuvant chemotherapy was employed in 196 (27%) patients, endocrine therapy in 261 (36%), and adjuvant radiation in the majority (559 [77.5%]).

### Survival analysis

Median follow-up for those without recurrence was 7.1 years (0.02–16.7), and median follow-up by the reverse Kaplan-Meier method for those with recurrence was 7.4 years (5th–95th percentiles 3.2–12.3). There were a total of 85 events of recurrence or death: 40 patients had recurrence and died, 23 had recurrence only, and 22 died without recurrence. Forty-one patients (65%) with recurrence had recurrence at a distant site first (13 [31.7%] brain only, 4 [10%] bone only, 4 [10%] liver only, 3 [7%] lung only, and 17 [41%] mixed/other). Nine of the 13 brain-only metastases occurred in HER2 amplified patients.

**Fig. 2:**
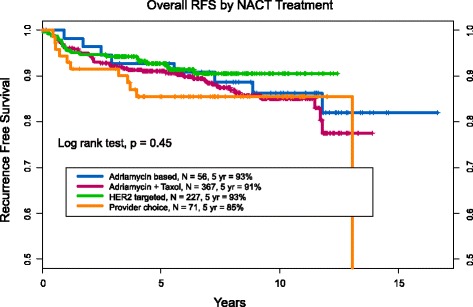
Recurrence-free survival for the entire cohort, compared between the four neoadjuvant chemotherapy (NACT) treatment regimens

There was no statistically significant difference in RFS by treatment group (Fig. 2) (log-rank test *P* = 0.45). Patients treated with A no T (*n* = 56) had a 5-year RFS of 93% (95% CI 86–100%), A + T (*n* = 367) 91% (88–94%), AT + HER2 (*n* = 227) 93% (89–96%), and PC (*n* = 71) 85% (78–94%) (Table 2, Fig. 2). Patients who were HER2+ who achieved a pCR and received HER2-targeted therapy had 5-year RFS similar to those who did not (94% received versus 90% no HER2 therapy; *P* = 0.19).

**Table 2:** Recurrence-free survival for the total cohort and by tumor subtype

Treatment group	Overall (N = 721, *P* = 0.45)		HR (*n* = 143, *P* = NA)		HER2-amplified (*n* = 312, *P* = 0.42)		TN (*n* = 216, *P* = 0.59)	
	No. of patients	5-yr RFS (%)	No. of patients	5-yr RFS (%)	No. of patients	5-yr RFS (%)	No. of patients	5-yr RFS (%)
A no T	56	93 (86–100)		–^a^		–^a^	22	86 (73–100)
A + T	367	91 (88–94)	124	92 (87–97)	55	89 (80–98)	170	92 (87–96)
AT + HER2	227	93 (89–96)		–^a^	220	93 (90–97)		–^a^
PC	71	85 (78–94)	11	80 (58–100)	33	91 (81–100)	21	85 (72–100)

*Abbreviations: HR* Hormone receptor, *TN* Triple-negative breast cancer, *A no T* Adriamycin-based therapy alone, *A + T* Adriamycin plus taxane, *AT + HER2* HER2-targeted therapy, *PC* provider choice

^a^Missing cells < 10 patients

**Fig. 3:**
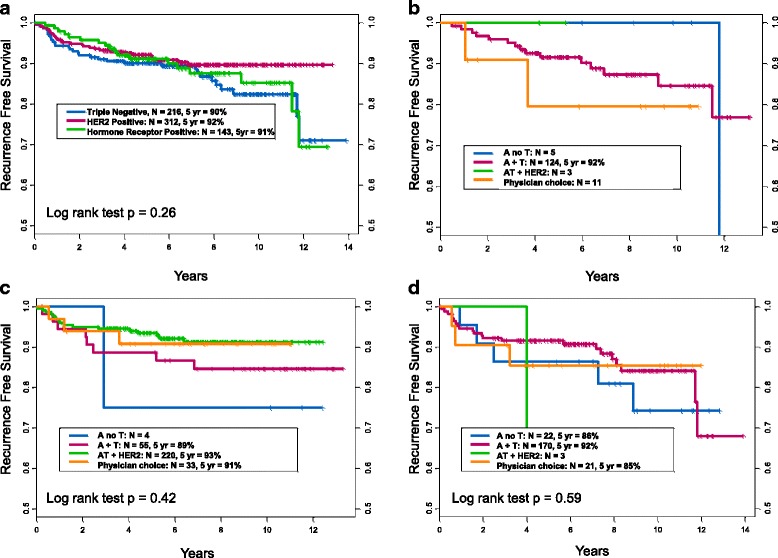
**a** Recurrence-free survival (RFS) by tumor subtype. **b** RFS by neoadjuvant chemotherapy (NACT) treatment regimen within hormone receptor (HR)-positive, HER2-nonamplified patients. **c** RFS by NACT treatment regimen within HER2-positive patients. **d** RFS by NACT treatment regimen within triple-negative patients with breast cancer. Some groups were too small to analyze, and survival could not be calculated (**b**, **c**, **d**). Additionally, the log-rank test could not be performed for HR-positive disease (**b**), owing to small sample size

By tumor subtype, HR-positive patients (*n* = 143) had a 5-year RFS of 91%, HER2-amplified patients (*n* = 312) 92%, and TN (*n* = 216) 90% (*P* = 0.26). Patients who had HER2+ disease had similar survival by HR status (94% HR+ versus 92% HR−; *P* = 0.12). Within each tumor subtype, RFS by NACT regimen was compared. There was no significant difference between significant NACT regimen within HER2-amplified disease (*P* = 0.42) or TN disease (*P* = 0.59) (Table 2, Fig. 3). The NACT regimen sample sizes within HR-positive disease were too small to accurately calculate the log-rank test.

**Fig. 4:**
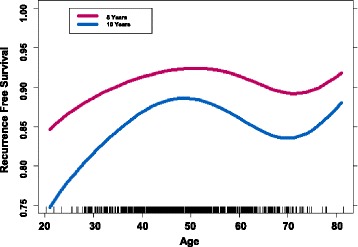
Recurrence-free survival by age as a continuous variable. There is low survival among very young patients

By unadjusted analyses, the RFS of 21- to 40-year-olds (*n* = 154) was 89%, 41- to 60-year-olds (*n* = 449) was 93%, and 61- to 90-year-olds (*n* = 118) was 88% (Fig. 4). RFS by stage was 93% for stage IIA patients (*n* = 292), 91% for stage IIB (*n* = 153), 95% for stage IIIA (*n* = 78), 86% for stage IIIB (*n* = 66), and 87% for stage IIIC (*n* = 132). RFS for grades 1 and 2 was 86% (*n* = 101), and for grade 3 (*n* = 599) it was 92%. Patients who were treated with ≤ 4 months of NACT had a 94% 5-year RFS, those treated with > 4 and ≤ 7 months 92%, and those treated with > 7 months 86% RFS (Additional file 1: Figure S1).

### Multivariate analysis

According to the 6-month landmark analyses, there were no differences attributable to adjuvant chemotherapy (92% with or without), endocrine therapy (91% without, 94% with endocrine therapy), or radiation treatment (92% with or without). Amongst 21- to 40-year-olds, RFS with A no T was 95% (*n* = 19), A + T 87% (*n* = 81), AT + HER2 88% (*n* = 43), and PC (*n* = 11) (too small to calculate). Amongst 41- to 60-year-olds, RFS was 90% for A no T, 93% A + T, 94% AT + HER2, and 88% PC (*n* = 32, 225, 149, and 43, respectively). In patients aged > 60 years, RFS was 88% in patients treated with A + T (*n* = 61) and 94% in patients treated with treated with AT + HER2 (*n* = 35). There were too few patients in the A no T and PC regimens to calculate survival (*n* = 5 and 17, respectively).

Adjusted RFS hazard ratios comparing each treatment with A + T were 1.25 (95% CI 0.47–3.35) for A no T, 0.90 (95% CI 0.37–2.20) for AT + HER2, and 1.28 (95% CI 0.55–2.98) for PC (Table 3). For age, the hazard ratio of death, compared with the reference group of 41- to 60-year-olds, was 2.00 (95% CI 1.05–3.82, *P* = 0.036) for 21- to 40-year-olds, and 2.04 for 61- to 90-year-olds (95% CI 1.01–4.15, *P* = 0.048). There was a significantly increased risk of death for stage IIIC patients (2.26, 95% CI 1.11–4.61, *P* = 0.024). There was no significant difference between the hazard ratio of death by menopausal status (*P* = 0.57); grade (*P* = 0.22); tumor subtype (HER2-amplified vs TN, *P* = 0.17; HR-positive vs TN, *P* = 0.99); or use of adjuvant chemotherapy (*P* = 0.45), adjuvant hormonal therapy (*P* = 0.40), or adjuvant radiation (*P* = 0.18). For NACT duration, the shortest duration (≤ 4 months) had survival similar to those with more conventional lengths of treatment (1.0, 95% CI 0.5–2.0 versus > 4 and ≤ 7 patients, *P* = 0.89), but patients with longer durations of NACT did worse (> 7 versus > 4 and ≤ 7, HR 1.9, 95% CI 1.1–3.3, *P* = 0.018) (Additional file 1: Figure S1).

**Table 3:** Hazard ratio of death, based on patient characteristics and treatments received

Variable	Contrast	HR (95% CI)	*P* value
Multivariate landmark analysis (*n* = 642)			
Regimen	AT + HER2 vs. A + T	0.90 (0.37–2.20)	0.82
	PC vs. A + T	1.28 (0.55–2.98)	0.56
	A no T vs. A + T	1.25 (0.47–3.35)	0.66
Age	21–40 vs. 41–60	2.00 (1.05–3.82)	*0.036*
	> 60 vs. 41–60	2.04 (1.01–4.15)	*0.048*
Postmenopausal	Yes vs. no	0.82 (0.43–1.59)	0.57
Stage	IIB vs. IIA	1.15 (0.60–2.23)	0.67
	IIIA vs. IIA	0.73 (0.24–2.15)	0.56
	IIIB vs. IIA	2.06 (0.87–4.88)	0.099
	IIIC vs. IIA	2.26 (1.11–4.61)	*0.024*
Grade	III vs. I/II	0.67 (0.35–1.28)	0.22
Subtype	HER2-amplified vs. TN	0.56 (0.25–1.28)	0.17
	HR vs. TN	1.00 (0.46–2.22)	0.99
Adjuvant chemotherapy	Yes vs. no	1.33 (0.64–2.77)	0.45
Adjuvant hormone therapy	Yes vs. no	0.75 (0.39–1.45)	0.40
Adjuvant radiation treatment	Yes vs. no	0.65 (0.34–1.22)	0.18

*Abbreviations: A no T* Adriamycin-based therapy alone, *A + T* Adriamycin plus taxane, *AT + HER2* HER2-targeted therapy, *PC* Provider choice, *HR* Hormone receptor, *TN* Triple-negative breast cancer

Italicized values are statistically significant

Boldface type represents those statistically significant values

## Discussion

It is well established that pCR differs by therapeutic regimen and breast cancer subtype [3, 6, 14, 23], with patients who achieve a pCR demonstrating improved survival over those who do not. However, any influence of the NACT regimen employed on subsequent survival in patients who have already achieved a pCR has not been explored previously. Data presented in the present study suggest that subsequent RFS among patients achieving pCR is not influenced by or associated with the regimen of NACT used to achieve that pCR. The very high recurrence-free survival in this population supports the value of achieving a pCR, but it also suggests that there may be limited value in continued systemic treatments in the postoperative setting for the pCR patient [15].

Because reaching a pCR, rather than the specific therapies used to achieve it, appears to be paramount, the early determination of treatment response will be an important research focus. Imaging alone [24–26] or targeted biopsy techniques [27, 28] may be effective tools in determining pCR after NACT. Imaging modalities that show promise in the assessment of chemotherapy response include ultrasound of both breast and axilla [24], dynamic contrast-enhanced magnetic resonance imaging (MRI) [29], MRI texture analysis [25], and trimodal imaging (ultrasound, mammography, and MRI) [30, 31]. Shear-wave elastography of tumor stiffness before the start of chemotherapy has been associated with tumor response [26], and ^18^F-fluorodeoxyglucose positron emission tomography/computed tomography (PET/CT) has also been studied [32]. The latter work correlated metabolic response by PET/CT during NACT after as few as two cycles of chemotherapy to improved survival at 71-month follow-up. Such imaging may allow oncologists to determine who is responding to chemotherapy earlier and tailor treatment accordingly or even avoid surgery altogether [33, 34]. Such concepts are being explored through the NRG BR005 [31] trial and a National Institutes of Health Breast Cancer Steering Committee initiative [35].

The generalizability of a single-institution cohort to NACT patients treated with current regimens or outside the institution requires confirmation. First, these data lack molecular phenotypes. It is known that luminal A tumors are less sensitive to chemotherapy [1], but the relationship of residual tumor and survival holds true across constructed subtype [3], so the presented work should be negligibly disadvantaged by this. Second, although there was no statistical difference in RFS between NACT regimens, the wide CIs do not demonstrate true equivalence between regimens. This may reflect the relatively low event rate of recurrences after pCR (85 of 721 patients), similar to recent trial reports [7, 9, 15], or it may be due to sample size. Third, it is difficult to interpret the effect of chemotherapy duration, owing to the retrospective nature of the analysis. NACT durations > 7 months are likely due to NACT complications and unexpected delays of surgical treatment, but the shorter-duration NACT patients present an interesting observation. Although it is promising that patients who achieved a pCR after only four cycles had survival similar to those receiving more conventional regimens, many patients at our institution are treated on study protocols or clinical trials, so this may be attributed to observational bias and, as such, should be interpreted cautiously. Regimen durations should be compared in future de-escalation trials. Last, these data were collected before taxotere/cyclophosphamide (TC) or paclitaxel+trastuzumab +/- pertuzumab (TH+/-P) were widely recognized as accepted neoadjuvant regimens, as indicated by the very few patients receiving these regimens, and highlighting the need now for further prospective trials to investigate the safe de-escalation of NACT.

Another consideration in this body of literature is that the relationship between NACT, pCR, and improved survival is complex. For example, the addition of taxanes led to increased pCR in NACT trials and also increased survival in adjuvant trials, suggesting an association between increased pCR and improved survival [36, 37]. However, the largest prospective cohort, NSABP B27, failed to show OS or DFS benefit despite a doubling of pCR. This influenced the pooled CTNeoBC analysis [1], which did not validate pCR as a surrogate endpoint for survival, suggesting that survival data after pCR should be interpreted with care.

Despite these limitations, this study does point to “at-risk” patient populations. Patients aged 21–40 years and stage IIIC patients had significantly increased risk of death on multivariate analysis. These higher-risk populations should be considered when making treatment decisions regarding additional adjuvant chemotherapy, and they merit future studies. Also, this study highlights that regardless of how you achieve pCR or RCB 0 (1), the outcome is the same. If a reliable imaging modality can be developed to predict treatment response after only a few cycles of chemotherapy, we may be able to administer taxane and HER2-targeted therapies only, and avoid adriamycin-based therapy, for example. Prospective trials minimizing neoadjuvant cytotoxic agents based on interim findings suggestive of pCR may be warranted.

## Conclusions

These data suggest that postsurgical RFS among patients with pCR is not significantly influenced by the type of NACT prior to surgery. Because this work provides indirect data only, meta-analysis of randomized trial data should be explored to evaluate wider populations and larger sample sizes to minimize bias. However, the present study provides some reassurance for designing prospective trials aimed at personalization and de-escalation of neoadjuvant chemotherapy.

## Additional file

Additional file 1: **Figure S1.** Five-year RFS by duration of neoadjuvant chemotherapy. (PPTX 72 kb)
