# Supplementary figures and images for: Effect of neoadjuvant chemotherapy regimen on relapse-free survival among patients with breast cancer achieving a pathologic complete response: an early step in the de-escalation of neoadjuvant chemotherapy
Source: Breast Cancer Res. 2018 Apr 16;20:27. doi: 10.1186/s13058-018-0945-7 (PMC5902970; doi:10.1186/s13058-018-0945-7)

## Slide 1
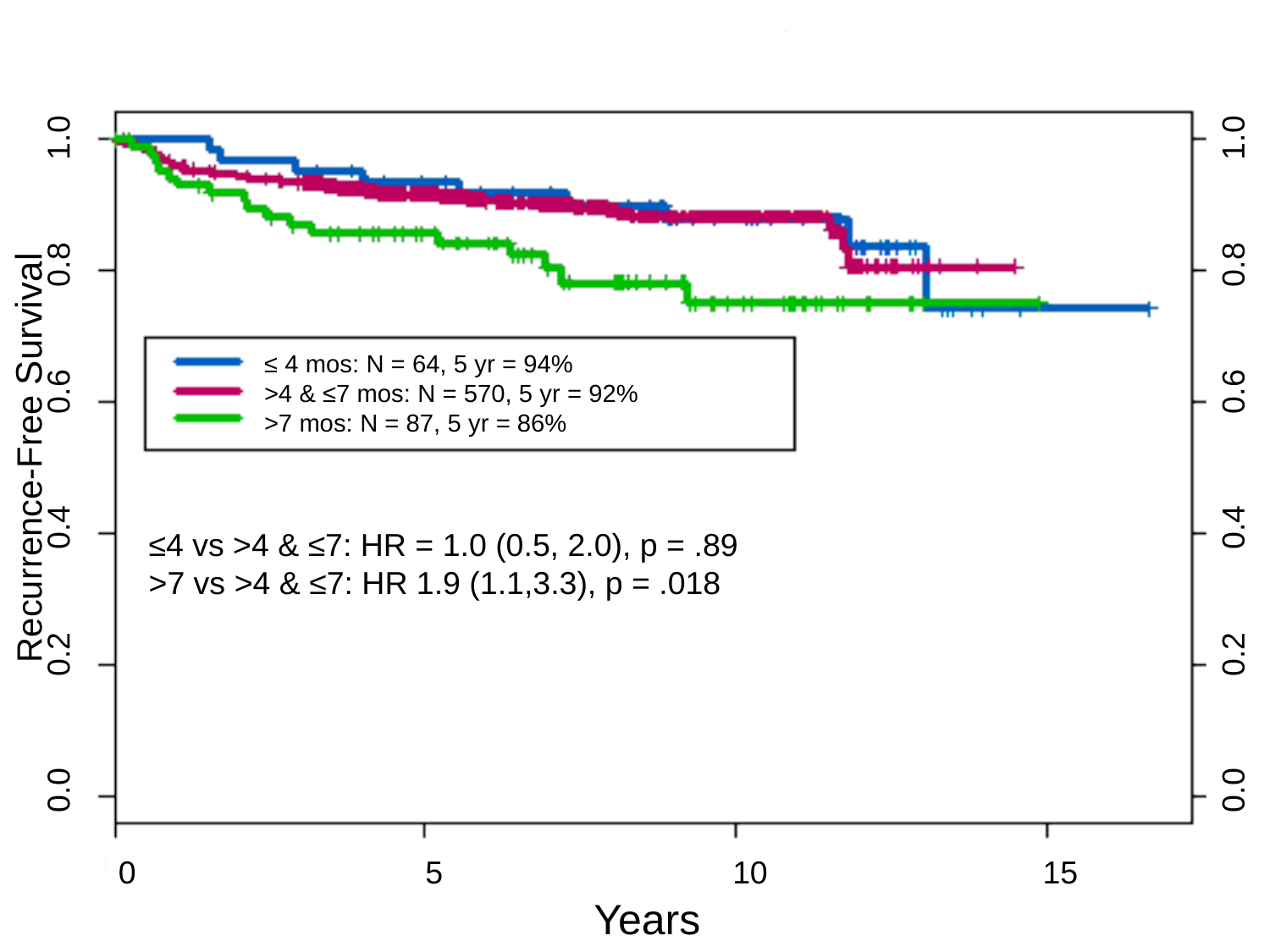

0.0	 0.2	 0.4	 0.6	 0.8	 1.0
0.0	 0.2	 0.4	 0.6	 0.8	 1.0
≤ 4 mos: N = 64, 5 yr = 94%
>4 & ≤7 mos: N = 570, 5 yr = 92%
>7 mos: N = 87, 5 yr = 86%
Recurrence-Free Survival
≤4 vs >4 & ≤7: HR = 1.0 (0.5, 2.0), p = .89
>7 vs >4 & ≤7: HR 1.9 (1.1,3.3), p = .018
0		 5		 10		 15
Years

Supplement: Supplementary file 1 — Figure S1. Five-year RFS by duration of neoadjuvant chemotherapy. (PPTX 72 kb) [file 13058_2018_945_MOESM1_ESM.pptx]
